# Factors Influencing Reproductive Performance in Austrian Sow Farms Challenged by Reproductive Disorders

**DOI:** 10.3390/vetsci13010003

**Published:** 2025-12-19

**Authors:** Gertrude Baumgartner, Alexander Grahofer, Andrea Buzanich-Ladinig, Christine Unterweger

**Affiliations:** 1 Unit for Swine Medicine, Clinical Department for Farm Animals and Food System Science, University of Veterinary Medicine, Veterinaerplatz 1, 1210 Vienna, Austria; andrea.ladinig@vetmeduni.ac.at (A.B.-L.); christine.unterweger@vetmeduni.ac.at (C.U.); 2 Clinic for Swine, Department for Clinical Veterinary Medicine, Vetsuisse Faculty, University of Bern, Bremgartenstrasse 109a, 3001 Bern, Switzerland; alexander.grahofer@unibe.ch

**Keywords:** reproductive disorders, breeding, farrowing rate, return-to-estrus, treatments, biosecurity, swine, management

## Abstract

Many piglet-producing farms are facing poor reproductive performance, yet their characteristics remain poorly understood. This study aimed to identify factors contributing to reproductive disorders based on information easily identifiable through an oral survey. Therefore, forty Austrian piglet producing farms (35–2000 sows) were surveyed using a questionnaire mainly addressing management, treatment and prophylaxis. Five key performance indicators (KPIs) were defined to assess farm productivity: farrowing rate, return-to-estrus rate, abortion rate, total piglets born per litter, and piglets weaned per litter. Farrowing rates were significantly higher in larger farms (r = 0.368; *p* = 0.019), PRRS-negative farms (r = 0.415; *p* = 0.008), and farms that did not clean the vulva before artificial insemination (r = 0.357; *p* = 0.024). Return-to-estrus rates were significantly higher in smaller farms (r = −0.431; *p* = 0.006), farms with more vaginal discharge after farrowing (r = 0.397; *p* = 0.011), those not using hormonal farrowing induction (r = 0.339; *p* = 0.033), and farms with older teaser boars (r = 0.385; *p* = 0.039). Larger farms had more piglets born per litter (r = 0.342; *p* = 0.031) and weaned more piglets (r = 0.391; *p* = 0.013). Weaned piglet numbers were also higher in farms with all-in/all-out implementation in farrowing pens (r = 0.353; *p* = 0.026), with restricted access of other animal species to the barn (r = 0.366; *p* = 0.028) and hormonal farrowing induction (r = 0.348; *p* = 0.028). Common well-established strategies for improving fertility performance like evaluation of body temperature after farrowing, antimicrobial, and hormonal treatments showed limited relevance. Further research is needed to confirm these findings and to identify additional factors influencing reproductive performance of sows.

## Simple Summary

Poor reproductive performance remains one of the most common and economically significant challenges in piglet-producing herds. A wide range of infectious but more frequently non-infectious influences—including herd management, genetic predisposition, and environmental stressors—can negatively affect sow fertility. The aim of this study was to identify easily assessable factors associated with reproductive disorders in sows by means of a structured oral survey. To this end, forty Austrian piglet-producing farms (35–2000 sows), all experiencing ongoing reproductive issues, were visited and evaluated using a comprehensive questionnaire addressing key aspects of herd management, housing, farrowing management, and biosecurity. Several management-related factors were identified as being linked to impaired reproductive performance. These included overall herd size, the age and use of the teaser boar, and multiple biosecurity-related practices at both internal and external levels. The findings underscore that many influential factors are readily observable during routine farm visits and can therefore be targeted for improvement without the need for complex diagnostics. This study highlights the importance of systematic on-farm assessment to detect management shortcomings contributing to reproductive problems and supports the implementation of targeted, farm-specific interventions to enhance sow fertility.

## 1. Introduction

Poor reproductive performance in sow herds is a major concern for piglet-producing farms, is associated with substantial economic losses, and can be influenced by a vast number of factors [1]. It is a multifactorial issue, influenced by a wide range of infectious and, more frequently, non-infectious factors, including management deficiencies, genetic predispositions, inadequate housing conditions [2,3], environmental stressors [4], and nutritional imbalances [5], all playing a significant role in influencing the performance characteristics of sow herds.

Systematic collection and evaluation of sow performance data are widely recognized as essential tools for monitoring both reproductive efficiency and overall herd productivity [6,7]. These data facilitate the benchmarking of reproductive outcomes and help identify weaknesses as well as opportunities for improvement. Sow performance characteristics on farms with reproductive problems are typically characterized by low farrowing rate, high return-to-estrus rate, or high abortion rate [8]; total born piglets and weaned piglets per sow are also important relevant performance characteristics to evaluate herd productivity [9]. Farrowing rate and return-to-estrus rate are influenced by various management factors including semen quality, accuracy of estrus detection, hygiene practices during artificial insemination [9,10], timing [11,12] and frequency [13] of insemination, catheter handling techniques [14], lactation length [15], body condition and weight [16], parity [17], and farm size [18]. In contrast, abortions are more frequently associated with infectious pathogens [8]; but non-infectious causes such as intoxication, heat stress, and further stressors and fear have also been shown to negatively impact pregnancy maintenance [2].

Although individual risk factors have been widely studied, few studies have evaluated how these factors interact. A more holistic approach is needed to better understand the causes of reproductive problems in piglet production. This study aimed to identify potential factors contributing to reproductive disorders in piglet-producing farms in Austria with a particular focus on farm size, management practices, hygiene, biosecurity, the use of treatments, and preventive strategies. The findings offer valuable insights into weaknesses and provide guidance for improving biosecurity, management, and animal health.

## 2. Materials and Methods

All participants were informed about the aim and structure of the study and provided their consent for the publication of acquired data.

Forty Austrian piglet producing farms with poor reproductive performance were examined to collect data about their specific management, prophylaxis, and treatment protocols by conducting a face-to-face questionnaire and analyzing farm performance characteristics. Poor reproductive performance was defined as a farrowing rate < 85% over the past year, return-to-estrus rates > 10% within the last three months, or abortion rates > 10% over the last three months, based on the criteria set by Pozzi and Loris [8]. The recruitment of participating farms was facilitated by herd-attending veterinarians following a nationwide call across Austria. Data collection was conducted by a single investigator between September 2023 and June 2024.

The survey (Supplementary Material) included the following categories related to herd and reproductive management: (1) general farm data including herd size, genetic background, and production system classification, (2) reproductive farm management encompassing batch farrowing rhythm, weaning and breeding days, insemination frequency, and use of hormonal induction for parturition, (3) breeding management including the use of teaser boars, origin and storage of semen, personnel performing artificial insemination (AI), pre-AI hygiene practices (e.g., vulvar cleaning), AI methodology, multiple or single use of AI catheters, and hormonal synchronization protocols, (4) health status and performance indicators addressing common herd health issues, sow replacement strategies, reproductive disorders, and overall sow performance data, Porcine Reproductive and Respiratory Syndrome (PRRS) status (5) farrowing management practices such as monitoring of postpartum rectal temperature and vaginal discharge, percentage of sows treated for postpartum dysgalactia syndrome (PPDS), (6) diagnostics, therapeutic and prophylactic measures including, vaccination protocols, antibiotic treatment and treatment protocols for reproductive disorders and PPDS, and (7) internal and external biosecurity examining the source of gilts, quarantine measures, presence of other animal species on farm, rodent/bird/fly control, hygienic entrance area, animal flow, dedicated protective clothing and shoes, and procedures for pen cleaning and disinfection.

Median farm size in Austria is 119 pigs [19]; therefore, farms were divided into four size categories of ten farms each: small farms (<81 sows), medium-sized farms (81–120 sows), large farms (121–180 sows), and very large farms (>180 sows). Quarantine measures were defined as correct if they complied with Austrian legal requirements [19,20]. Farms’ PRRS statuses were classified as negative or positive [20,21]. Cleaning of sow’s vulva prior to AI was defined as correct if a new, dry, and clean paper towel was used for each individual sow and as incorrect if no or wet cleaning was performed.

All data were systematically transferred to a spreadsheet program (Microsoft Office^©^ Excel 2016) and subjected to statistical analysis using IBM SPSS statistics software (Version 29.0.1.0 (171)). Due to the sample size (*n* = 40), Lillifors-corrected Kolmogorov–Smirnov test was employed to assess the normality of distribution for continuous variables. Depending on the distribution, either the Pearson correlation coefficient (for normally distributed data) or the Spearman rank correlation coefficient (for non-parametric data) was applied to examine associations between management factors and reproductive parameters. Eta coefficient and regression analysis were used for correlations between metric and nominal data. For all statistical tests, a confidence interval of 95% was set and statistical significance was considered at a *p*-value < 0.05, and *p*-value > 0.05, but <0.10 is described as a trend. Correlations and associations were investigated between the above-mentioned herd management practices and five key performance indicators (KPIs) which were defined as (1) farrowing rate within the last year, (2) return-to-estrus rate within the last three months, (3) abortion rate within the last three months, (4) the number of total piglets born within the last year, and (5) piglets weaned per litter within the last year.

## 3. Results

### 3.1. General Information of the Sow Farms

Farms were located in regions of high pig density within upper Austria (*n* = 15), lower Austria (*n* = 13), and Styria (*n* = 12) and were supervised by 18 different herd-attending veterinarians. Herd sizes ranged from 35 to 2000 sows (median 121.5 sows). All farms conducted batch farrowing with production rhythms varying between one and five weeks. While differences in KPIs depending on production rhythm were found, no statistically significant correlations were identified (Table 1).

Among all farms, 22 operated as farrow-to-finish systems (integrating piglet production and finishing on the same farm), whereas 18 specialized exclusively in piglet production, selling pigs at a weight of 25–30 kg to external finishing units. Three farms operated under certified organic production systems, but displayed a production intensity comparable to that of the other farms included in the study. Gilt replacement strategies varied as follows: 75% of the farms sourced gilts from external suppliers, whereas 25% produced their gilts on-site.

Digital data management programs were used on 82.5% of the farms to manage reproductive data. In contrast, 17.5% of farmers recorded all data manually, using paper lists, index cards, or relying on memory.

### 3.2. Reproductive Performance Characteristics

**Figure 1:**
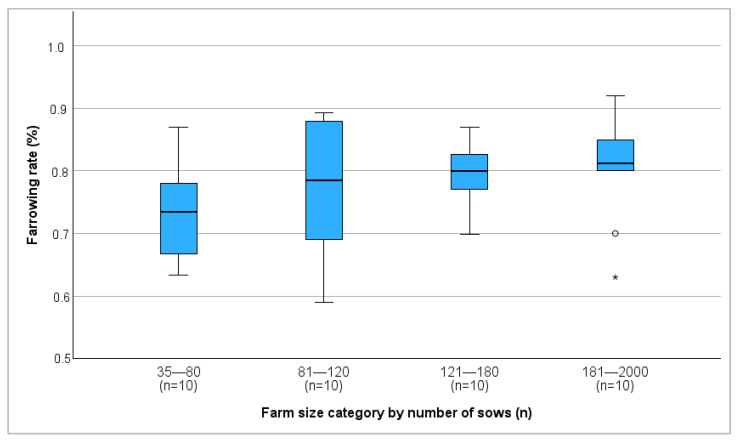
Median farrowing rate of different farm size categories in percent. °: outliers, values are more than 1.5 times the interquartile range away from the box. *: extreme outliers.

An overview on the reproductive performance parameters is provided in Table 2 and illustrated in Figure 1. The median parity of sows before being removed from farms was 6 (min: 3.5; max: 11.7), no significant correlation with KPIs was found. The main reason for culling sows was reproductive failure (57.5%) followed by old age/poor performance (35.0%), lameness (5.0%), and genetic improvement (2.5%).

**Figure 2:**
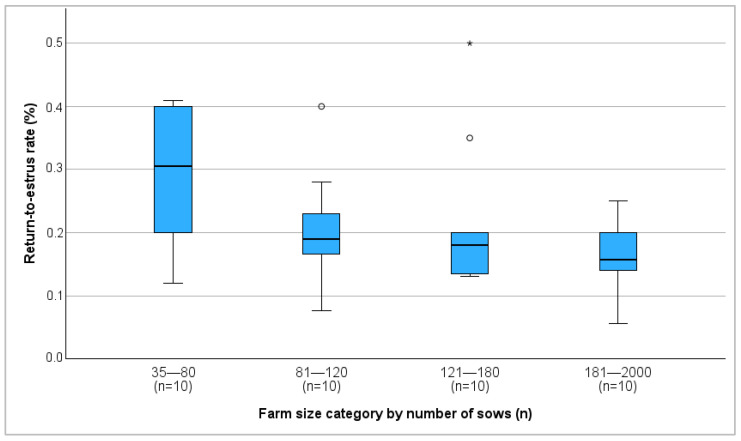
Median return-to-estrus rate on different farm size categories in percent. °: outliers, values are more than 1.5 times the interquartile range away from the box. *: extreme outliers.

Farm size and farrowing rate showed a significant positive correlation (r = 0.368, *p* = 0.019), with the average farrowing rate increasing as farm size increased (Figure 1). In contrast, farm size was negatively correlated with the return-to-estrus rate (r = −0.431, *p* = 0.006) (Figure 2). No correlation between farm size and abortion rate was detected.

**Figure 3:**
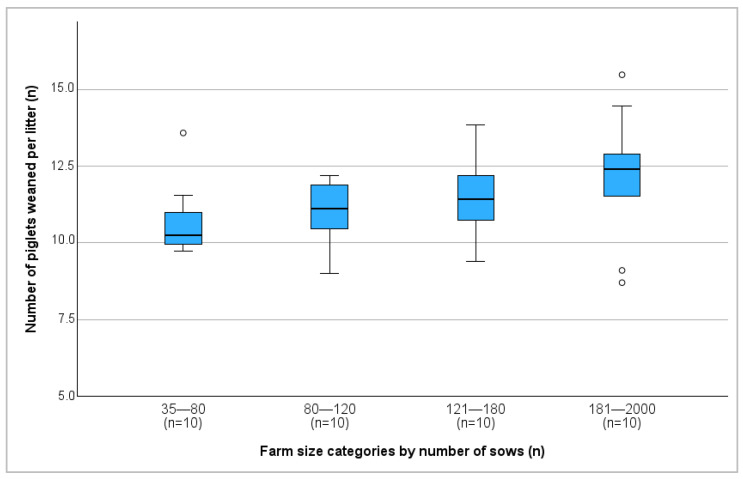
Number of piglets weaned per litter on different farm size categories. °: outliers, values are more than 1.5 times the interquartile range away from the box.

Farm size exhibited a significant positive correlation with both the total number of piglets born (r = 0.342; *p* = 0.031) and the number of piglets weaned per litter (r = 0.391; *p* = 0.013) (Figure 3).

### 3.3. Management Factors

#### 3.3.1. Measures Taken for Farm Staff Hygiene

All farms required personnel to change into designated farm-specific clothing prior to entry. In addition, 35% of farms provided dedicated footwear for different areas. A hygienic entrance area equipped with hand-washing facilities was present on 80% of the farms. Showers were present on 30% of farms; however, only 10% reported consistent use of these facilities.

#### 3.3.2. Animal Flow

Proper quarantine procedures were implemented on 38.5% (*n* = 10) of farms that sourced gilts externally. However, no correlations were found between proper quarantine and KPIs. The all-in/all-out (AIAO) principle was applied in 82.5% of farrowing rooms and in 77.5% of nurseries. On 5% of farms, AIAO was not implemented at any production stage. A separate room for breeding was available on 52.5% of farms, whereas the remaining farms used multipurpose rooms for both breeding and gestation. Dynamic group housing during gestation was practiced in 12.5% of farms, 87.5% had fixed groups, and no differences in KPIs were found. On 25% of farms, nursery or grower pigs had direct or indirect contact to sows, either through partially shared corridors or shared air space. Farms that had implemented AIAO in farrowing rooms weaned significantly more piglets per litter (median: 11.5 vs. 10; r = 0.353, *p* = 0.026). Those farms also had higher median numbers of total piglets born (median: 14.7 vs. 14; r = 0.283, *p* = 0.077) and lower return-to-estrus rates (median: 18% vs. 30%; r = 0.279; *p* = 0.081).

#### 3.3.3. Cleaning and Disinfection Practices in Farrowing Rooms

High-pressure washers were utilized on farms for efficient and thorough cleaning of equipment, infrastructure, and surfaces. On half of the farms, farrowing pens were cleaned after each batch using hot or cold water in combination with soap. An additional 47.5% of farms cleaned farrowing pens using water, hot or cold, alone, without the application of detergents. One farm did not clean farrowing pens regularly. Disinfection following cleaning was implemented on 75% of the farms. No significant correlations with KPIs were found.

#### 3.3.4. Other Animals on Farm

**Figure 4:**
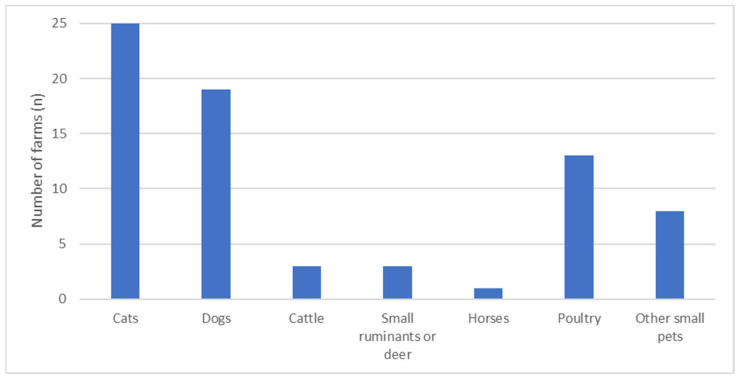
Overview of the number of farms with livestock animals and non-agricultural animals and other small pets such as rabbits, hamsters, and guinea pigs on site.

Birds were observed inside pig barns on 47.5% of farms. Problems with rodent activity were reported by 87.5% of farmers, while 22.5% reported considerable problems with fly infestations. However, no significant correlations with the KPIs were identified. In total, 90% (*n* = 36) of farms housed not only pigs but also other livestock and pets (Figure 4). For 62.5%, companion animals, primarily cats or dogs, had access to the pig barn. Farms that allowed other animal species to enter the pig barn weaned significantly less piglets per litter (median: 10.8 vs. 12.2; r = 0.366, *p* = 0.028).

### 3.4. Breeding Management

Semen used for AI was either purchased from external boar studs (65%), derived from the farm’s own boars (15%) or a combination of both sources (20%). On 75% of farms (*n* = 34), a designated semen storage box was available. However, no statistically significant correlations were found between the presence of a semen storage box and any of the KPIs. The number of individuals performing AI per farm ranged from one to four. Specifically, one person conducted AI on 40% of farms, two individuals on 50%, three on 7.5%, and four on 2.5% of farms. The number of inseminators did not correlate with KPIs. According to farmer reports, sows detected in estrus were inseminated a median of 2.2 times (min: 1.7; max: 3.1) per estrus. No significant correlation between the number of inseminations and KPIs was observed. The number of boars used for estrus detection and/or breeding varied from one to five per farm. Average number of boars increased with farm size.

Age data for boars were available from 29 farms. The median (teaser) boars’ age was 2.5 years with two boars reaching up to 10 years of age. The age of the teaser boars correlated with the farm’s return-to-estrus rate (r = 0.385; *p* = 0.039). No other significant correlations with KPIs were found. No significant correlations were found between semen source and KPIs.

Natural breeding, in addition to AI, was practiced on five of 40 farms (12.5%). Two farms did not use a boar for estrus detection, and subsequently not for breeding. Both were within the lower 50 percentile of farrowing rates. On farms where natural breeding was performed, the median number of total piglets born was 14.18 and median piglets weaned per litter was 9.96. In contrast, on farms that exclusively relied on AI (*n* = 35), the median values were 14.5 for total piglets born and 11.3 for piglets weaned. Correlations were not significant (r = 0.268; *p* = 0.095) and no other correlations with KPIs were found.

**Figure 5:**
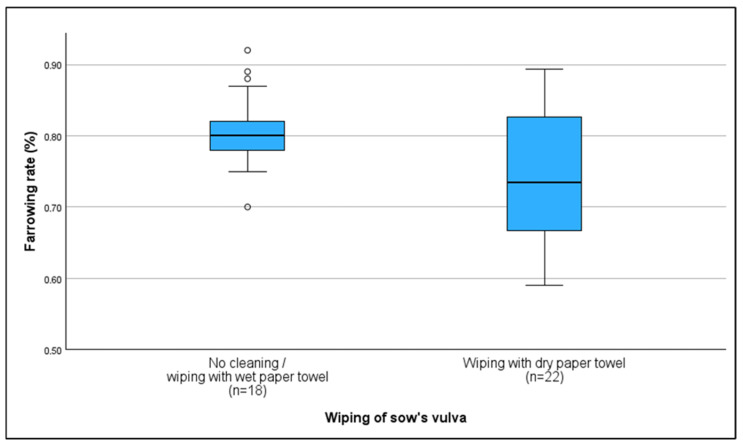
Average farrowing rate based on vulva cleaning protocols before artificial insemination; correct: wiping with dry paper towel; incorrect: no cleaning/wiping with wet paper towel. °: outliers, values are more than 1.5 times the interquartile range away from the box.

Vulvar hygiene practices prior to AI varied among farmers. The majority of farmers (67.5%) cleaned the sow’s vulva before breeding by wiping it with either a wet (10%) or dry paper towel (55%). Farms either cleaning with a wet towel or without any cleaning measures were found to have higher farrowing rates (80%); (r = 0.357; *p* = 0.024) compared to those cleaning with a dry paper towel (73.5%). No other significant correlations with KPIs were found (Figure 5).

### 3.5. Management of Farrowing

In total, 90% of the farrowing units were equipped with conventional farrowing crates, while 10% utilized free-farrowing pens. On 40% of farms, pre-farrowing hygiene measures included washing sows, either prior to entering the farrowing unit or within the pen itself. On 50% of farms, farrowing was hormonally induced using prostaglandin F2α (PGF2α).

Farms on which farrowing was hormonally induced were more likely to have stillbirth rates below 10% (r = 0.327, *p* = 0.028; median stillbirth rate: 7.4% vs. 8.3% in the other farms). In addition, these farms exhibited a tendency toward higher farrowing rates (median: 80% vs. 76.5%; r = 0.307, *p* = 0.054), significantly lower return-to-estrus rates (median: 16.65% vs. 21.5%; r = 0.339, *p* = 0.033), and weaned a higher number of piglets per litter (median: 11.84 vs. 10.93; r = 0.348, *p* = 0.028). No other significant correlations with KPIs were found.

**Figure 6:**
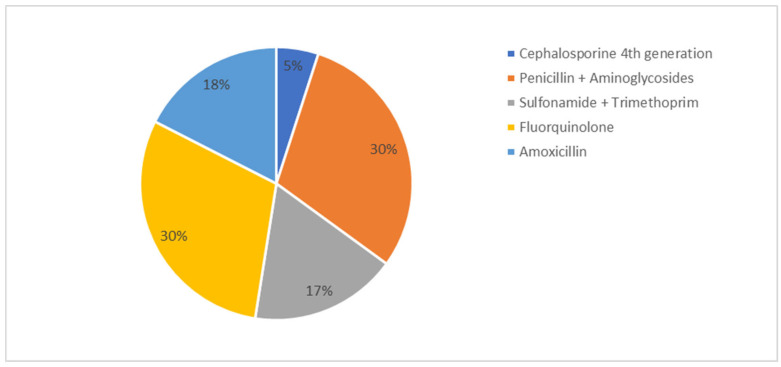
Classes of antibiotics and combinations of these used for treatment of postpartum dysgalactia syndrome (PPDS) in sows in percent of farms.

Monitoring of rectal body temperature post-farrowing was conducted on 25% of farms, but this practice did not correlate significantly with KPIs. The median proportion of sows treated for PPDS was 9.2%, with a wide inter-farm variation (range: 1–50%). Treatment strategies for PPDS included the use of fluoroquinolones and combinations of penicillin and aminoglycosides, as reported by the farmers (Figure 6). Nonsteroidal anti-inflammatory drugs (NSAIDS) were applied on 87.5% (*n* = 35) of farms in case of PPDS, while oxytocin was used on 17.5% of farms. No significant correlations between percentage of sows affected by PPDS, choice of antimicrobial treatment, use of NSAIDS or oxytocin with KPIs were found.

The median percentage of sows with purulent vaginal discharge after farrowing was 5.3. A positive correlation was observed between the prevalence of vaginal discharge and return-to-estrus rate (r = 0.397; *p* = 0.011), indicating that farms with more sows exhibiting vaginal discharge after farrowing also had higher return-to-estrus rates. Additionally, this was negatively correlated with the abortion rate (r = −0.328; *p* = 0.039).

### 3.6. Pharmaceutical Interventions for Stimulating the Estrus Cycle

**Figure 7:**
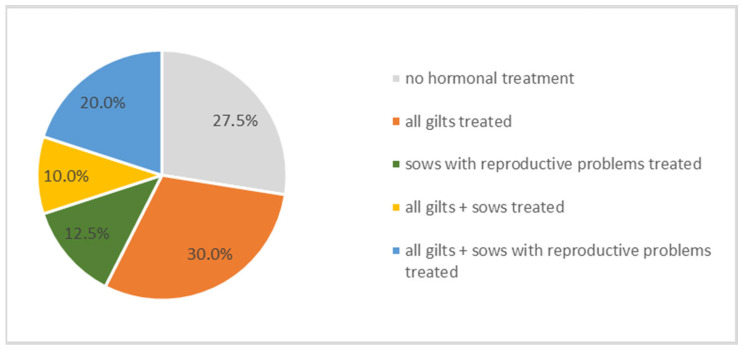
Percentage of farms treating animal groups with hormones for stimulating the estrus cycle.

Hormonal treatments for estrus induction or cycle synchronization were employed on a subset of the farms. Specifically, 30% of farms utilized hormonal protocols to induce estrus in gilts, while 12.5% administered hormonal treatments to sows exhibiting reproductive disorders. Conversely, 27.5% (*n* = 11) of farms reported no use of hormonal interventions for estrus induction or synchronization (Figure 7). Farmers stated using pharmaceutical products containing altrenogest, serum-gonadotropin, and/or choriongonadotropin. However, no significant correlations were identified between the use of hormonal estrus induction or synchronization protocols and KPIs.

### 3.7. Antibiotic Treatment of Reproductive Problems

**Figure 8:**
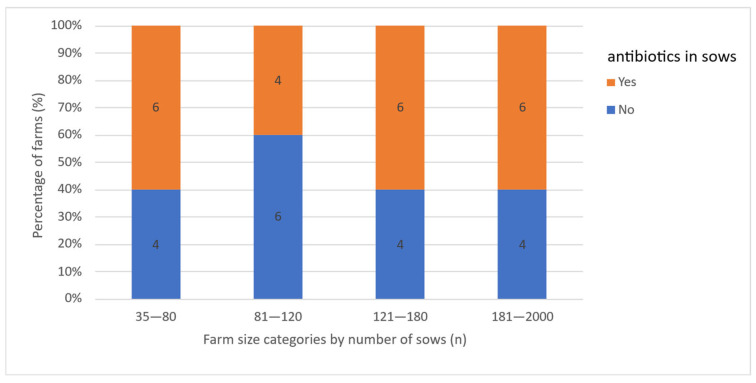
Percentage of farms that did or did not use antibiotics for reproductive failure, by farm size category; numbers within the bars represent absolute values.

**Figure 9:**
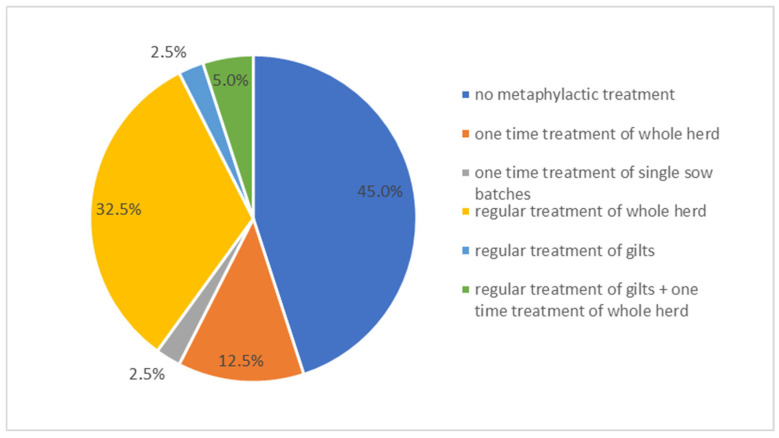
Percentage of farms using different treatment methods for sows with reproductive problems.

In case of reproductive performance issues, 55% (*n* = 22) of farmers reported the use of antimicrobials either at the group or herd level, regardless of farm size category (Figure 8). Twenty farms used tetracyclines and two farms used macrolides. Among these 22 farms, 73% (*n* = 16) had implemented antibiotic treatment on a regular basis for the last two years as treatment for increased return-to-estrus rates (*n* = 14) or abortion rates (*n* = 2). Twenty-seven percent (*n* = 6) used antimicrobial treatment as a one-time intervention in response to acute reproductive issues, specifically elevated return-to-estrus rates (*n* = 5) or abortion rates (*n* = 1) (Figure 9).

**Figure 10:**
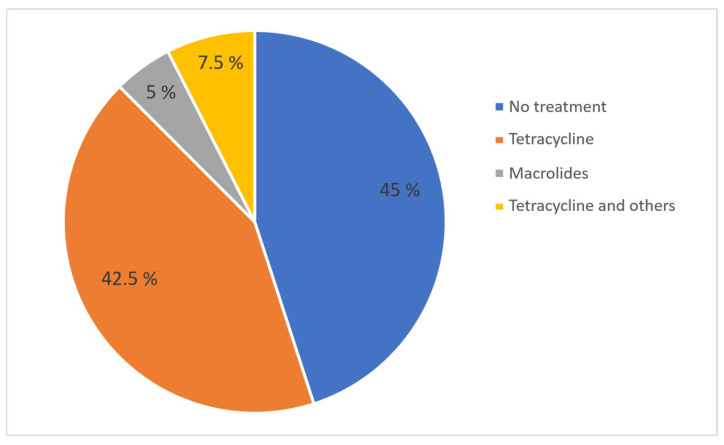
Percentage of farms using different antibiotic classes for treatment of reproductive problems in sows.

**Figure 11:**
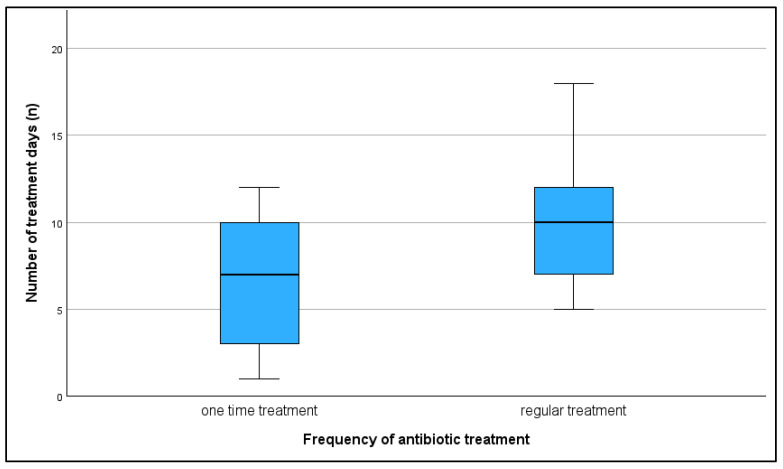
Number of days of antibiotic treatment in farms which treated their sows once and farms with regular treatment.

The use or non-use of antimicrobials, as well as regular versus one-time use, did not show a significant correlation with any of the KPIs. However, a trend was observed indicating that farms with regular antimicrobial use tended to have lower farrowing rates compared to farms with one-time use (r = 0.410; *p* = 0.058). The median farrowing rate on farms with regular antimicrobial use was 80%, on farms with one-time use 85.5%, while on farms with no antimicrobial treatment the median farrowing rate was 75.5%. A total of 45% of farms did not administer antibiotic treatment regularly to sows despite the presence of reproductive issues. Also, 42.5% of farms used tetracyclines, 5% used macrolides, and 7.5% used tetracycline in combination with other antibiotics to treat reproductive disorders in sows (Figure 10). All treatments, except for one, were administered orally once a day. The number of treatment days ranged from 1 to 18, with a median of 9.5 days. On average, farms with regular treatments were found to have more treatment days per treatment, but this was not found to be significant (r = 0.351; *p* = 0.109) (Figure 11). Treatments started post-weaning and continued through the breeding period depending on the complete number of treatment days.

### 3.8. Vaccinations Against Reproductive Disorders

Vaccination protocols targeting reproductive diseases were implemented on all farms, with either all gilts or all sows vaccinated accordingly (Table 3). Different vaccination protocols were implemented against reproductive diseases. For Porcine Reproductive and Respiratory Syndrome (PRRS), all herds used a modified live virus (MLV) vaccine.

### 3.9. PRRS Status and Further Diagnostics

Twenty percent (*n* = 8) of farms had a negative PRRS status and exhibited higher farrowing rates (median: 84.3% vs. 77.5%; r = 0.415; *p* = 0.008) in comparison to PRRS positive herds. Five PRRS negative farms were large farms (120–180 sows) (Table 4).

Diagnostics, like vaginal swabs, blood sampling, or sampling abortion material to investigate reproductive problems were conducted on 50% of farms within the last year, according to the farmers. Among farms using antimicrobial treatment, 54.5% performed diagnostics related to reproductive problems. Findings from the conducted diagnostics were Antibodies against *Chlamydiaceae* (*n* = 7) and/or *Leptospira* spp. (*n* = 2), Influenzavirus antibodies (*n* = 2), PRRSV (*n* = 2), or no findings (*n* = 7); and *Chlamydiaceae* antigen in abortion material (*n* = 1).

## 4. Discussion

This study aimed to identify management-related factors contributing to reproductive problems in Austrian piglet-producing farms using an oral survey. While effective in gathering practical insights, this method may have overlooked key influencing factors such as housing conditions, semen quality [22], body weight and condition, feed ratios and nutrient level differences in gestation and lactation feeds among tested farms [6], and mycotoxins [23,24]. Additionally, due to the sample size (*n* = 40) and smaller scale of Austrian farms [25,26,27], some findings (e.g., batch farrowing, housing conditions, and family-owned farms) may have limited applicability internationally. Although not all participating farmers used digital data management programs, sufficient productivity records were available on each farm to calculate KPIs.

Larger herds tended to show better reproductive performance, likely due to more professionalized management and breeding practices, consistent with previous studies [18,27]. Larger herd size has been linked to higher efficiency [18] and owners of larger herd sizes may have better education and training in breeding management, contributing to an improved breeding success [28].

Farms without implementation of AIAO in farrowing pens showed significantly lower weaning rates and trends toward lower total born and higher return-to-estrus rates, likely due to insufficient hygiene and increased disease pressure in both sows and piglets. Although no significant correlations between KPIs and washing methods or disinfection of farrowing pens were found, sufficient washing and disinfection can only be conducted in an empty stable. Therefore, it is advisable to wash and disinfect the empty farrowing pen after each farrowing batch. Furthermore, to prevent manure buildup around the perineal region, flores should be kept as clean as possible; thus, measures such as scraping the floor and stalls are recommended [23]. Similarly, access of other animals to the pig barn was associated with reduced average weaning performance, possibly reflecting lower biosecurity levels and a higher risk of pathogen introduction. For example, leptospirosis can be introduced by rodents as well as by dogs and cats [7]. Consequently, the entry of other animals to the pig barn needs to be avoided and presence of other animals in the barn could serve as an indicator of overall biosecurity level on the farm.

Farms with negative PRRS status had significantly higher farrowing rates, likely due to lower pathogen burden and better biosecurity and management, supporting previous findings [29]. Most PRRS free farms were in the size category “large”, being in line with the findings that larger farms often have better management [27]. However, PRRS directly influences reproductive performance of sows [30].

The number of median inseminations per estrus on each farm (min: 1.7; max: 3.1) did not correlate with the observed KPIs. Although improvements in fertility through multiple inseminations have been reported [7], proper timing of the last insemination within estrus is considered more critical, as insemination too late in estrus can reduce fertility [31]. Timing should therefore be evaluated at the herd level, taking into account the weaning-to-estrus interval and estrus signs [32].

Older boars were associated with increased return-to-estrus rates, aligning with evidence of declining semen quality and libido in aging boars due to low testosterone levels [33,34] or a potentially more harmful semen microbiota [35]. In addition, breed differences, seasonal effects, and reproductive health have all been reported to affect libido in boars [36,37,38]. Therefore, whether used as teaser or mating boars, it is crucial to continuously monitor both libido, semen quality, and health status to ensure high reproductive performance of sow herds. For farms using their own boars, maintaining hygiene during semen collection is vital to prevent contamination and preserve semen quality [39]. While most farms stored semen in temperature-controlled conditions [40], no significant performance differences were observed, possibly due to fast usage or alternative cool storage [41,42]. Proper hygiene during AI is crucial to prevent bacterial introduction into the genital tract [23,43,44]. Clean hands, single-use catheters [40], and disposable gloves [23] are essential when performing AI in sows. While dry cleaning of vulva is seen as correct, and is recommended for improved hygiene [23,40], this study found that reproductive performance was poorer on farms where this method was used. This was also found by Young et al. (2010), who speculated that the continuous use of the same cleaning tissue for each sow could result in the transfer of pathogens, as seen in cattle cleaning practices [45,46]. However, in this study, every farmer stated to use single-use wet tissue or paper towels only. Anyway, improper cleaning of vulva can decrease farrowing rate [10], as it is possible that by improper cleaning techniques fecal particles may be introduced into the genital tract rather than being removed. Proper vulva hygiene should be regarded as crucial.

Puerperal disorders, particularly PPDS [47,48] and endometritis [49,50], multifactorial diseases with highly variable clinical symptoms [51], were variably prevalent and managed with different treatment regimens. As parturition is a crucial time point in sow health, close monitoring is crucial [47]. Biomarkers such as abnormal vaginal discharge and elevated body temperature have proven useful for early detection [49,52]. Vaginal discharge post-partum correlated with higher return-to-estrus rates after weaning, suggesting ongoing uterine infection [23,49]. Seemingly contradictory to this statement is the negative correlation of vaginal discharge and abortion rate, as ongoing infections can later lead to abortions. However, it is probable that sows showing vaginal discharge post-farrowing, with ongoing uterus infections, are not able to become pregnant in the first place, which would explain an increasing effect on the return-to-estrus rate and a decreasing effect on the abortion rate.

Although monitoring inner body temperature post-farrowing is recommended, it did not correlate with KPIs. However, all farmers employed visual or haptic and behavioral assessments such as observing the sows’ feed intake, nursing behavior, and mammary glands to assess sow health. This suggests that both objective and subjective monitoring methods are valuable in evaluating the health status of breeding sows in the puerperium.

Only 87.5% of farmers stated using NSAIDs in their treatment protocols for PPDS. Although no correlations with treatment protocols and KPIs were found, the usage of NSAIDs is recommended for treating PPDS due to their anti-inflammatory and anti-endotoxemic effects, while the use of antimicrobials should be evaluated on a case-by-case basis. However, oxytocin is recommended in all cases to induce milk ejection [7,53]. As performance characteristics and PPDS are interlinked [47,54], prioritizing sow’s health after farrowing as well as piglet health is essential.

No significant difference in reproductive performance was observed between sows with reproductive disorders that received antimicrobial treatment and those that did not. However, a trend showed lower farrowing rates on farms with regular antibiotic treatments, with a 10% lower average farrowing rate compared to one-time treatment. While this may suggest that metaphylactic antimicrobial treatment is necessary to prevent further declines in performance, it also underscores that chronic reproductive disorders, being multifactorial in nature, cannot be effectively addressed solely through routine antimicrobial use. As diagnostics were lacking in 50% of farms, implementing adequate treatment and prophylactic measures was challenging. Moreover, the lack of targeted diagnostics limited the ability to emphasize the potential development of antimicrobial resistance in pathogens commonly associated with genital infections, such as *Streptococcus* spp. [55], *Chlamydia* [56,57], *Leptospira* [58], or *Escherichia coli* [59]. Higher antimicrobial usage in sows is also associated with higher use in piglets form birth to slaughter [60], further indicating that excessive and regular antibiotic treatment veils underlying management problems but cannot solve them.

## 5. Conclusions

The findings of this study underscore the importance of targeted management practices to improve reproductive outcomes in Austrian piglet-producing farms. Simple but consistent measures—such as implementing AIAO systems, maintaining strict biosecurity, and ensuring proper hygiene during AI—can significantly enhance fertility parameters. Regular evaluation of boar health, careful monitoring of sows during the puerperal phase, and judicious use of hormones and medications are essential tools for optimizing sow productivity. While some practices showed only statistical trends, their biological relevance should not be overlooked. Importantly, reproductive performance cannot be improved by isolated interventions alone but requires a holistic, well-adapted management strategy. Farms that prioritized health, hygiene, and supervision at critical time points, like mating, farrowing, and puerperium, consistently performed better. This highlights the need for continuous education, disease prevention, and refinement of farm-specific protocols. Future studies should integrate objective performance data and broader environmental factors to build on these insights.

## Figures and Tables

**Table 1:** Differences in key reproductive parameters based on production rhythm.

Production Rhythm (Weeks)	*n*		Farrowing Rate (%)	Return-To-Estrus Rate (%)	Abortion Rate (%)	Live Born Piglets/Sow/Litter	Weaned Piglets/ Sow/Litter
1	3	Min	63	10	0	13.20	8.70
		Max	85	20	4	19.45	15.49
		Median	80	16	4	16.46	12.30
		Mean	76	15	2	16.37	12.16
		SD	12	5	2	3.13	3.40
2	4	Min	60	8	1	12.00	9.10
		Max	88	40	20	16.60	14.46
		Median	75	21	1	14.67	11.15
		Mean	75	23	6	14.48	11.47
		SD	12	14	10	1.89	2.22
3	21	Min	59	11	0	10.73	9.00
		Max	92	50	9	16.50	12.20
		Median	79	20	1	14.18	11.00
		Mean	79	23	2	13.97	10.89
		SD	8	11	2	1.38	0.95
4	5	Min	80	13	1	14.24	11.23
		Max	83	18	3	16.50	13.39
		Median	81	15	2	15.45	12.50
		Mean	81	15	2	15.34	12.44
		SD	1	2	1	0.89	0.81
5	7	Min	65	6	0	13.50	10.00
		Max	88	41	12	19.35	13.86
		Median	70	20	1	14.60	10.80
		Mean	74	23	4	15.41	11.68
		SD	8	12	5	2.13	1.68

min: minimum, max: maximum, SD: standard deviation.

**Table 2:** Sow performance data in all 40 farms.

	Min	Max	Median	Mean	SD
Farrowing rate (%)	59	92	80	77	8
Return-to-estrus rate (%)	6	50	20	22	10
Abortion rate (%)	0	20	1	3	4
Litters/sow/year	1.84	2.53	2.26	2.24	0.16
Total born piglets/sow/litter	10.73	19.45	14.37	14.63	1.78
Live born piglets/sow/litter	10.43	17.03	13.13	13.21	1.48
Weaned piglets/sow/litter	8.7	15.49	11.27	11.38	1.51
Total weaned piglets/sow/year	17.92	36.4	25.45	25.52	4.28
Stillborn rate/year (%)	1.4	18.60	7.94	8.33	3.92
Replacement rate (%)	18	65	40	41	11

min: minimum, max: maximum, SD: standard deviation.

**Table 3:** Percentage of farms in which gilts and/or sows are vaccinated against diseases influencing reproductive performance.

Disease	Gilts	Sows
PRRS	77.5%	77.5%
Porcine Parvovirosis	100%	95%
Erysipelas	100%	95%
Leptospirosis	20%	20%
Influenza A	30%	27.5%
PCV2-RD	17.5%	7.5%

PRRS: Porcine Reproductive and Respiratory Syndrome, PCV2-RD: Porcine Circovirus 2-Reproductive Disease.

**Table 4:** PRRS status on different sized farms.

Farm Size Category	PRRS Status		Total
	Negative	Positive	
Smal (<81)	1	9	10
Medium (81–120)	1	9	10
Large (121–180)	5	5	10
Very large (>180)	1	9	10
total	8	32	40

## Data Availability

The original contributions presented in this study are included in the article and Supplementary Material. Further inquiries can be directed to the corresponding author.

## Supplementary Materials

The following supporting information can be downloaded at https://www.mdpi.com/article/10.3390/vetsci13010003/s1; Supplementary Material: Questionnaire for management factors.

## Abbreviations

The following abbreviations are used in this manuscript:

AI	Artificial insemination
AIAO	all-in/all-out
KPI	Key performance indicator
MLV	modified live virus
NSAIDS	Nonsteroidal anti-inflammatory drugs
PGF2α	Prostaglandin F2α
PPDS	Postpartum Dysgalactia Syndrome
PRRS	Porcine Reproductive and Respiratory Syndrome
