# Supplementary material for: Factors Influencing Reproductive Performance in Austrian Sow Farms Challenged by Reproductive Disorders
Source: Vet Sci. 2025 Dec 19;13(1):3. doi: 10.3390/vetsci13010003 (PMC12846600; doi:10.3390/vetsci13010003)
Supplement: Supplementary file 1 [file vetsci-13-00003-s001.zip › vetsci-4033866-supplementary.pdf]

Number: Date:

Farm Data

Name of farm:
Address:
Federal state:
Registration number:
Phone Number:

- Trial group:
    - Reproductive problems + tetracycline
    - Reproductive problems without tetracycline
- Number of sows:
- Genetics:
- Farm type:
    - Conventional
    - Organic
    - Farrow-to-finish farm
    - Piglet producer

1. Farm management

- Batch farrowing rhythm
    - 3 weeks
    - 4 weeks
    - Other..

- Weaning day
    - Mon
    - Tue
    - Wed
    - Thu
    - Fri
    - Sat
    - Sun

- Main breeding day
    - Mon
    - Tue
    - Wed
    - Thu
    - Fri
    - Sat
    - Sun

- How often are sows inseminated on average per estrus? ....

- Is hormonal induction of birth performed?
    - Yes...
    - No

Number: Date:

- Source of gilts
  - Gilts born and raised on farm
  - gilts from an external source
  - Other..

- Measures taken for quarantine
  - Own pen, same barn and air space as herd
  - Own pen, separate air space
  - Own stable = own pen in another building
  - No quarantine

- Number of days of quarantine:..

2. Breeding

- Teasing boar on duty?
  - Yes: age of boar...............................
  - no

- Origin of sperm
  - Boar stud
  - Other external source (e.g. neighbor)
  - Breeding boar
    - Natural breeding; age...
    - Artificial insemination; age.......

- How many persons do perform artificial insemination?...............................
- Temperate sperm cabin?
  - Yes
  - No

- Does the vulva get cleaned before artificial insemination?
  - Yes wet, water only
  - Yes wet, water + cleaning agent
  - Yes dry
  - no

- Which artificial insemination method is used?
  - Intrauterine
  - Intracervical (catheter with yellow head)

- Is the insemination catheter used more than once?
  - Yes...
  - No

Number: Date:

3. Health data and general problems

- Most common health problem on the farm (not fertility related)
  ....

- Does conjunctivitis occur frequently on the farm/is it treated?
  - No
  - In sows
  - In suckling piglets
  - In nursery piglets
  - In fattening pigs
  - Boars

- Replacement of sows
  - Replacement rate (%)...........................
  - Sows culled/year.........................
  - Most common reason for culling..........
  - Average age/number of litters at culling……….

- Name of slaughterhouse, where sows are culled
  ....

- For which animals is estrus hormonally induced?
  - None
  - Gilts
  - Sows
  - Animals with reproductive problems
  - All

- Which hormonal treatment protocol is used?
  - No synchronization
  - FSH/LH administration after weaning
  - Synchronization with Altrenogest
  - Synchronization with Altrenogest + FSH/LH

Number: Date:

4. Reproductive performance data:

! Have sow planner handed out!

Sow performance data of last 12 months

- Farrowing rate %...
- Return-to-estrus rate %..
- Abortion rate %...
- Number of non-productive-days..
- Litters/sow/year...
- Total number of piglets born/sow/litter..........................
- Number of piglets born alive/sow/litter.........................
- Number of weaned piglets/sow/litter.....................................
- Stillborn rate %...
- Mummies %...
- Gilts/sows differences if available ....

5. Reproductive problems:

- What is the main reproductive problem?
  - Abortions
  - Increase of Return-to-estrus rate
  - PPDS
  - SMEDI
  - Vaginal discharge
  - Other..

- How many sows show vaginal discharge after farrowing: ....%
- Description of discharge
  - Purulent
  - Mucous
  - Smelly
  - Reddish/brown

- Which parities (number of litters) are affected?
  - 1
  - 2
  - 3-5
  - 6-9
  - >9
  - Varies

- Is inner body temperature routinely measured in sows after farrowing?
  - Yes
  - No
- How many sows have an increased temperature after farrowing?...................... %
- How many sows show PPDS after birth…...................... ... %

Number: Date:

6. Diagnostics

- Which samples have been taken for diagnostics of reproductive problems?
  - o Abortion material
  - o Genital swabs
  - o Blood samples
  - o None

- Results of the diagnostics carried out?
  - o None
  - o ....

- Which diagnostics specific for *Chlamydia suis* have been performed?
  - o None
  - o Direct detection of antigen via PCR
    - - Material?..
  - o Indirect via antibody detection in blood samples
  - o Other..

Number: Date:

7. Use of medication and vaccinations

- PRRS status
  - Unsuspicious
  - Positive

8. Vaccination of pigs against reproductive disorders

Pathogene	Gilts	Sows	Vaccine
PRRS			
Porcine Parvovirosis			
Erysipelas			
Leptospirosis			
Influenza A			
PCV2-RD			

Metaphylactic antibiotic treatment

- Are antibiotics regularly administered to all animals or individual groups?
  - No metaphylactic antibiotic treatment at herd/group level
  - To the whole herd
  - To individual sow groups (e.g. during breeding)

Which treatment protocol was followed?

- Intervals
  - time based, e.g. 2x/year
  - Production-based, e.g. breeding batch
  - Other...............................
- Method of administration
  - Feed
  - Drinking water
  - Other...
- Name of the preparation...
- Dose/concentration..
- Number of days of administration:

Therapies

- Therapy for PPDS
  - Medications:..
  - Duration of application................................
  - Dose..
  - Application method...............................

- How are acute reproductive problems treated?
  ……………………………………………………………………..
- How long are antibiotics used?
  - No antibiotics used for fertility problems
  - Number of days:..
- Application?
  - Orally
  - Intramuscularly
- Who is treated?
  - Individual animals
  - Sow batches
  - Whole herd

Number: Date:

9. **Biosecurity, animal flow and other**
- Do other animals have access to the barn
  - No
  - Cats
  - Dogs
  - Small ruminants
  - Cattle
  - Other.................

- Other animals on the farm?
  - No, only pigs on the farm
  - Cats
  - Dogs
  - Cows
  - Small ruminants
  - Horses
  - Other.................

- Is there a problem with rodents/birds etc.?
  - Birds
  - Rats
  - Mice
  - Excessive flies

10. **Cleaning/disinfection in general, but especially in the breeding room and farrowing pen:**

- Are sows washed before farrowing?
  - Yes...
  - No

- All-in/all-out is implemented in which areas?
  - Nowhere on the farm
  - Farrowing pen
  - Gestation pen
  - Breeding pen
  - Quarantine room
  - Piglet area

- Cleaning of farrowing pen
  - How is cleaning done (water, soap, etc) ....
  - Cleaning with a high-pressure washer?
  - Disinfectant........................
  - How long is farrowing room left empty between groups....
  - Exposure time of disinfectant..................................
  -
- Cleaning of gestation pen:
  - How is cleaning done (water, soap, etc) ....
  - Cleaning with a high-pressure washer?
  - Disinfectant........................
  - How long is gestation pen left empty between groups...
  - Exposure time of disinfectant..................................

- Description of the entrance area:
  - Showering mandatory for external persons (vet)
  - ....
- Do weaned piglets have contact with sows via driveways or similar?
  - Yes..
  - No
- Are separate shoes used for different areas
  - Yes..
  - No

11. Notes:

Cleanlines, washing rooms, Change of shoes between piglets and sows, animal contact, production areas?

Overcrowding e.g. in nursery
